# Supplementary material for: Polymeric Microneedles for Transdermal Delivery of Rivastigmine: Design and Application in Skin Mimetic Model
Source: Pharmaceutics. 2022 Mar 30;14(4):752. doi: 10.3390/pharmaceutics14040752 (PMC9028500; doi:10.3390/pharmaceutics14040752)
Supplement: Supplementary file 1 [file pharmaceutics-14-00752-s001.zip › pharmaceutics-1618889-supplementary.pdf]

pharmaceutics

Supplementary Materials: Polymeric Microneedles for Transdermal Delivery of Rivastigmine: Design and Application in Skin Mimetic Model

Tânia M. T. Guimarães , Tânia Moniz , Cláudia Nunes , Maya Margaritova Zaharieva , Mila Kaleva , Krassimira Yoncheva , Hristo Najdenski , Sofia A. Costa Lima and Salette Reis

Table of Contents

	Page
**Figure S1 -** Experimental setup of skin irritation tests. …..……………......................	3
**Figure S2 -** Viscosity of the three different hydrogel formulations. ………………	3
**Figure S3 -** Representative SEM images of 400 and 800 µm drug-free MNs. ……	4
**Figure S4 -** Representative force-displacement curves of drug-free and calcein-loaded MNs made from alginate (left) or alginate/k-CRG (right). …………………	5
**Figure S5 -** Calcein permeation profile. …………………………………………...	6
**Figure S6 -** Apparent permeability coefficient for 3, 6, 8, and 24 hours of calcein for porcine ear skin treated with free calcein, calcein-loaded hydrogels, and calcein-loaded MNs of two different heights. ……………………………………	8
**Figure S7 -** Optical microscopy analysis of skin sample from the permeation assay. ………………………………………………………………………………..	9
**Figure S8 -** Representative force vs displacement curves for 800 µm drug-free and RV-loaded MNs. ………………………………………………………………..	9
**Figure S9 -** Skin rivastigmine permeation profile. ………………………………	10
**Figure S10 -** Apparent permeability coefficient for 3, 6, and 8 hours of RV for porcine ear skin treated with Exelon®, RV-loaded hydrogels, and RV-loaded MNs. ……………………………………………………………………………....	11
**Figure S11 -** Skin irritation test for alginate-MN and Mix-MN after 4 hours exposition. ……………………………………………………………………….	11
**Table S1 -** Scoring system for skin reaction. …………………………………….	12
**Table S2 -** PII or CII by rabbits. ………………………………………………….	12
**Drug release kinetics** …………………………………………………………….	12
**Table S3 -** Value of r2 obtained from the release data for different models of mechanism of drug release. ………………………………………………………	13

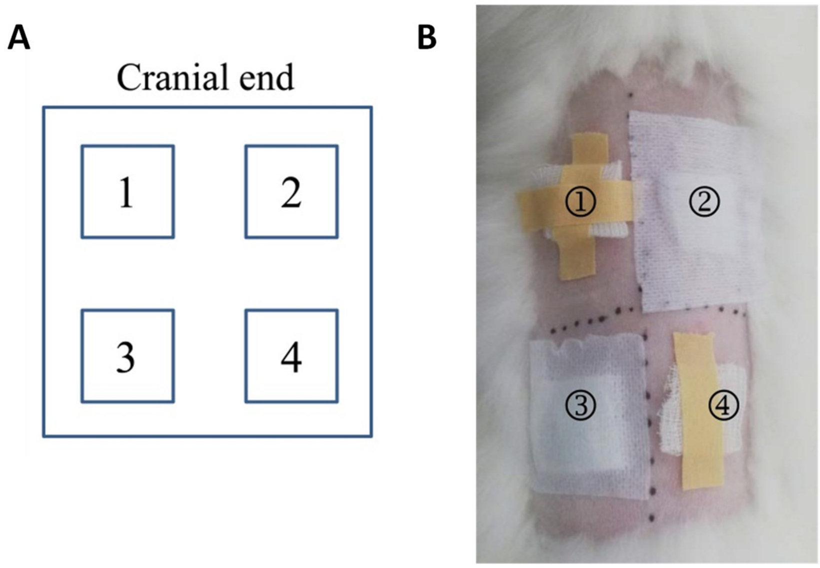

Figure S1. Experimental setup of skin irritation tests**.** (A) Location of test samples on the skin application sites: 1 - test site 1; 2 - positive control (10% SDS); 3 – Exelon® patch; 4 - test site 2. (B) General view of the experimental sites.

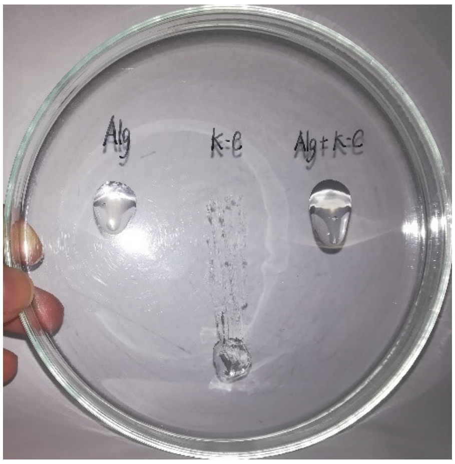

Figure S2. – Viscosity of the three different hydrogel formulations. Formulations were placed in a Petri dish, then the dish was tilted 90° and the hydrogel run was observed for 15 seconds. In the left is alginate 7% (w/w), in the middle k-CRG 1% (w/w) and in the right alginate/k-CRG (1:1) (w/w).

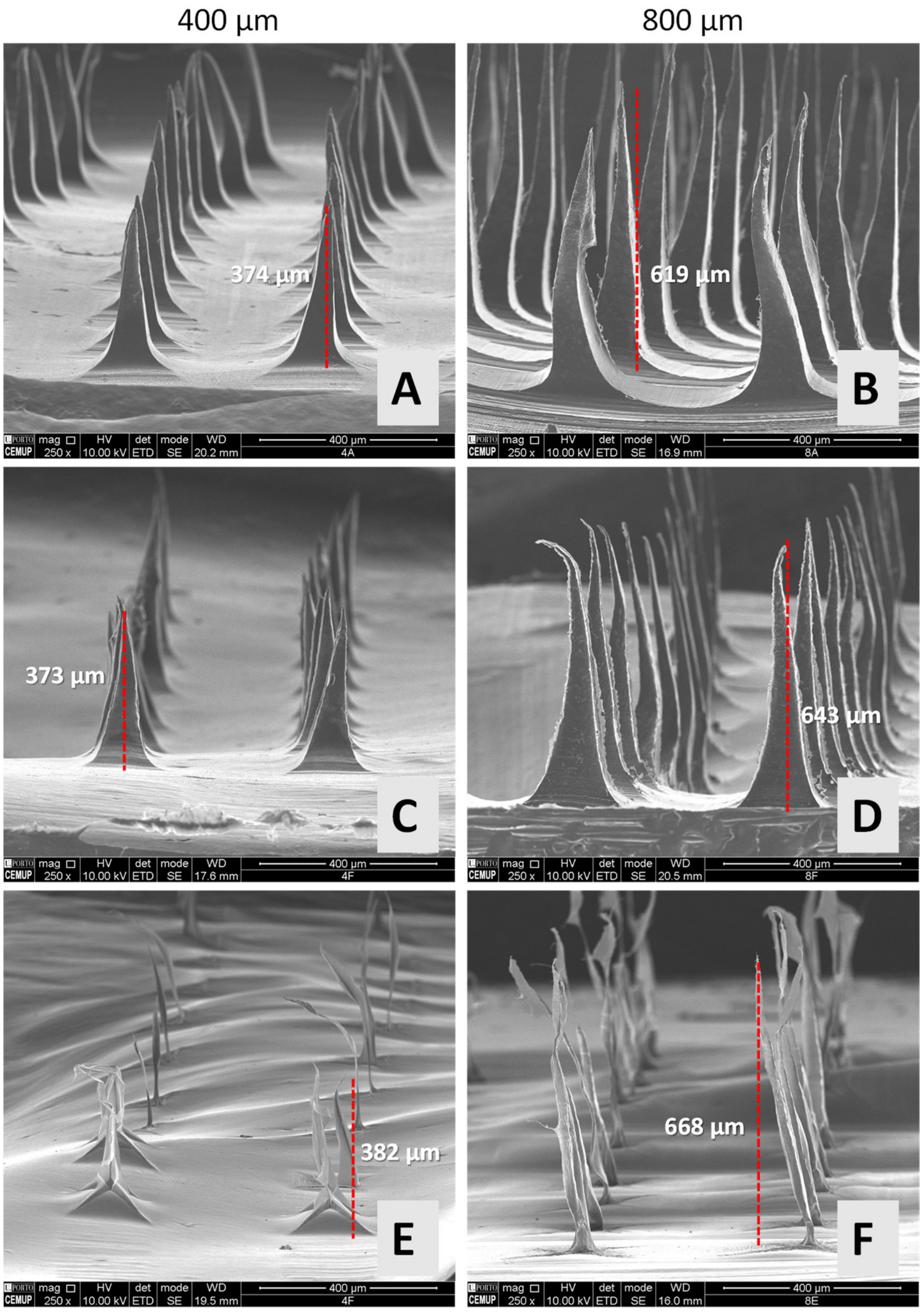

Figure S3. Representative SEM images of 400 and 800 µm drug-free MNs. (A) and (B) alginate MN; (C) and (D) alginate/k-CRG MN; (E) and (F) k-CRG MN.

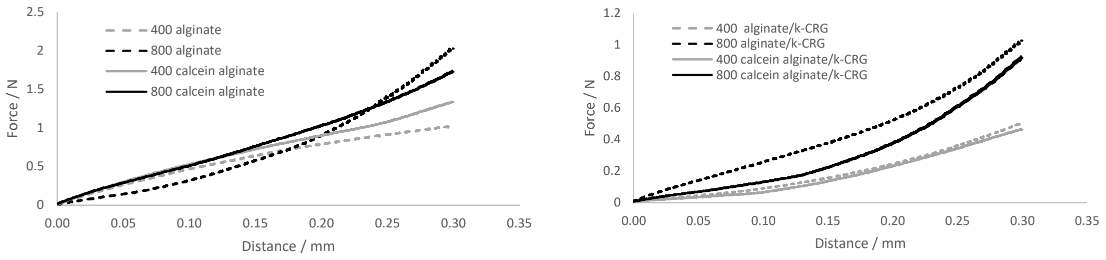

Figure S4. - Representative force-displacement curves of drug-free and calcein-loaded MNs made from alginate (left) or alginate/k-CRG (right).

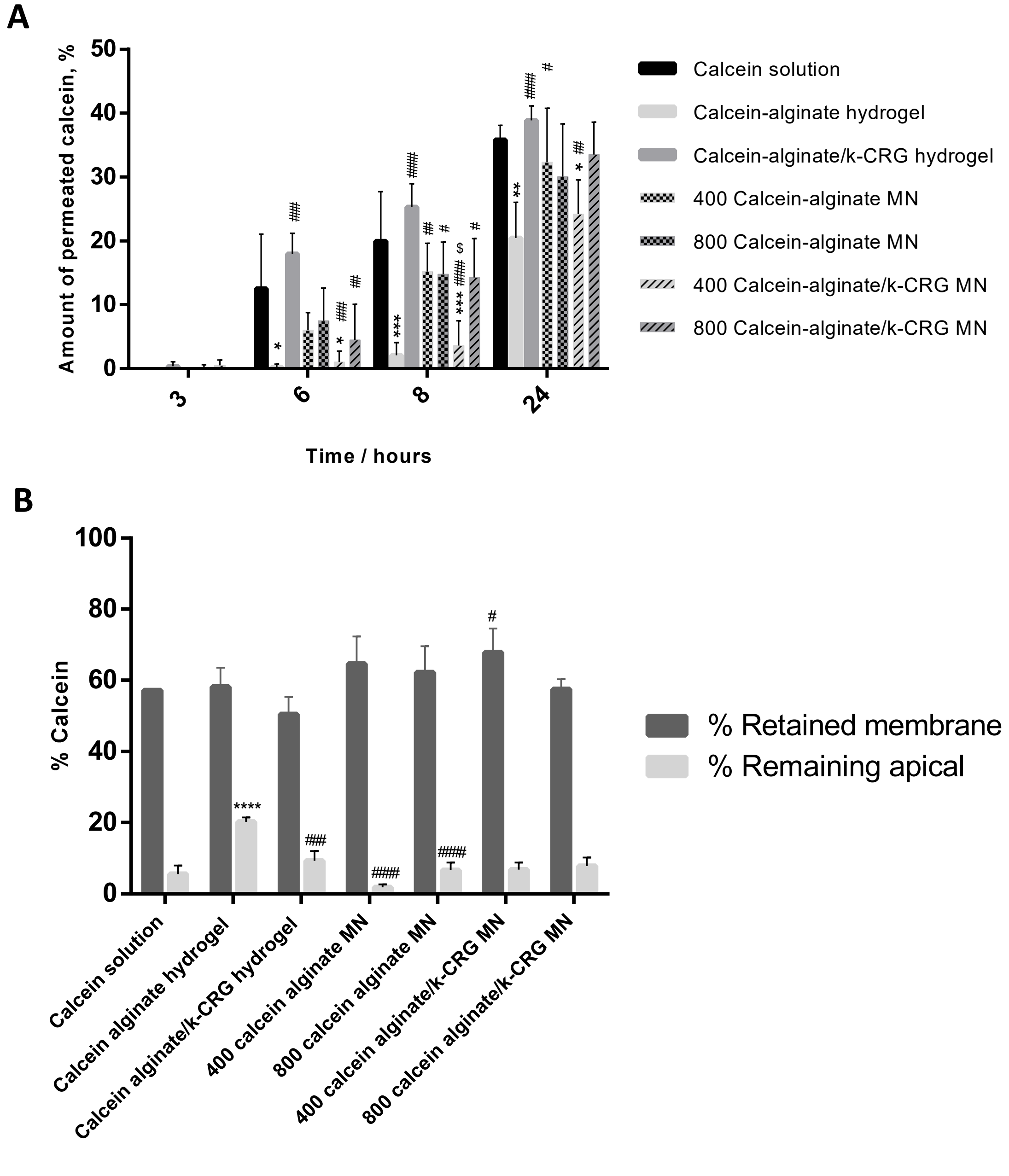

Figure S5. – Calcein permeation profile. The bars/points represent the mean ± SD of at least three independent experiments (n=3). (A) Percentage of permeation of calcein for porcine skin treated with free calcein, calcein-loaded hydrogels, and calcein-loaded MNs of two different heights, at different timepoints. $^{*}P < 0.05$ for calcein-loaded alginate hydrogel formulation and 400 calcein- alginate/k-CRG MN vs calcein solution at 6 hours; $^{*}P < 0.05$ for 400 calcein-alginate/k-CRG MN vs calcein solution at 24 hours; $^{**}P < 0.01$ for calcein-alginate hydrogel vs calcein solution at 24 hours; $^{***}P < 0.001$ for calcein-loaded alginate hydrogel formulation vs calcein solution at 8 hours; $^{***}P < 0.001$ for 400 calcein- alginate/k-CRG MN vs calcein solution at 8 hours; $^{\#}P < 0.05$ for 800 calcein-alginate MN and 800 calcein-alginate/k-CRG MN vs hydrogel formulation at 8 hours; $^{\#}P < 0.05$ for 400 calcein-alginate MN vs hydrogel at 24 hours; $^{\#\#}P < 0.01$ for 800 calcein-alginate/k-CRG MN vs hydrogel at 6 hours; $^{\#\#}P < 0.01$ for 400 calcein-alginate MN vs hydrogel at 8 hours; $^{\#\#}P < 0.01$ for 400 calcein-alginate/k-CRG MN vs hydrogel at 24 hours; $^{\#\#\#}P < 0.001$ for calcein-alginate/k-CRG hydrogel and for 400 calcein-alginate/k-CRG MN vs hydrogel at 6 hours; $^{\#\#\#\#}P < 0.0001$ for calcein- alginate hydrogel and 400 calcein-alginate/k-CRG MN vs hydrogel at 8 hours; $^{\#\#\#\#}P < 0.0001$ for calcein-alginate/k-CRG hydrogel vs hydrogel at 24 hours; $^{\$}P < 0.05$ for 400 calcein-loaded alginate/k-CRG MN vs 400

calcein-alginate at 8 hours. (B) Distribution of calcein among skin retained and non-permeated through the porcine skin after 24 hours. ****P < 0.0001 for calcein-alginate hydrogel vs calcein solution remaining in the apical; ###P < 0.001 for calcein-alginate/k-CRG hydrogel vs calcein-alginate hydrogel remaining in the apical; ####P < 0.0001 for 400 calcein-alginate MN and 800 MN vs hydrogel remaining in the apical; # P < 0.05 for 400 calcein-alginate/k-CRG MN vs hydrogel retained in the skin.

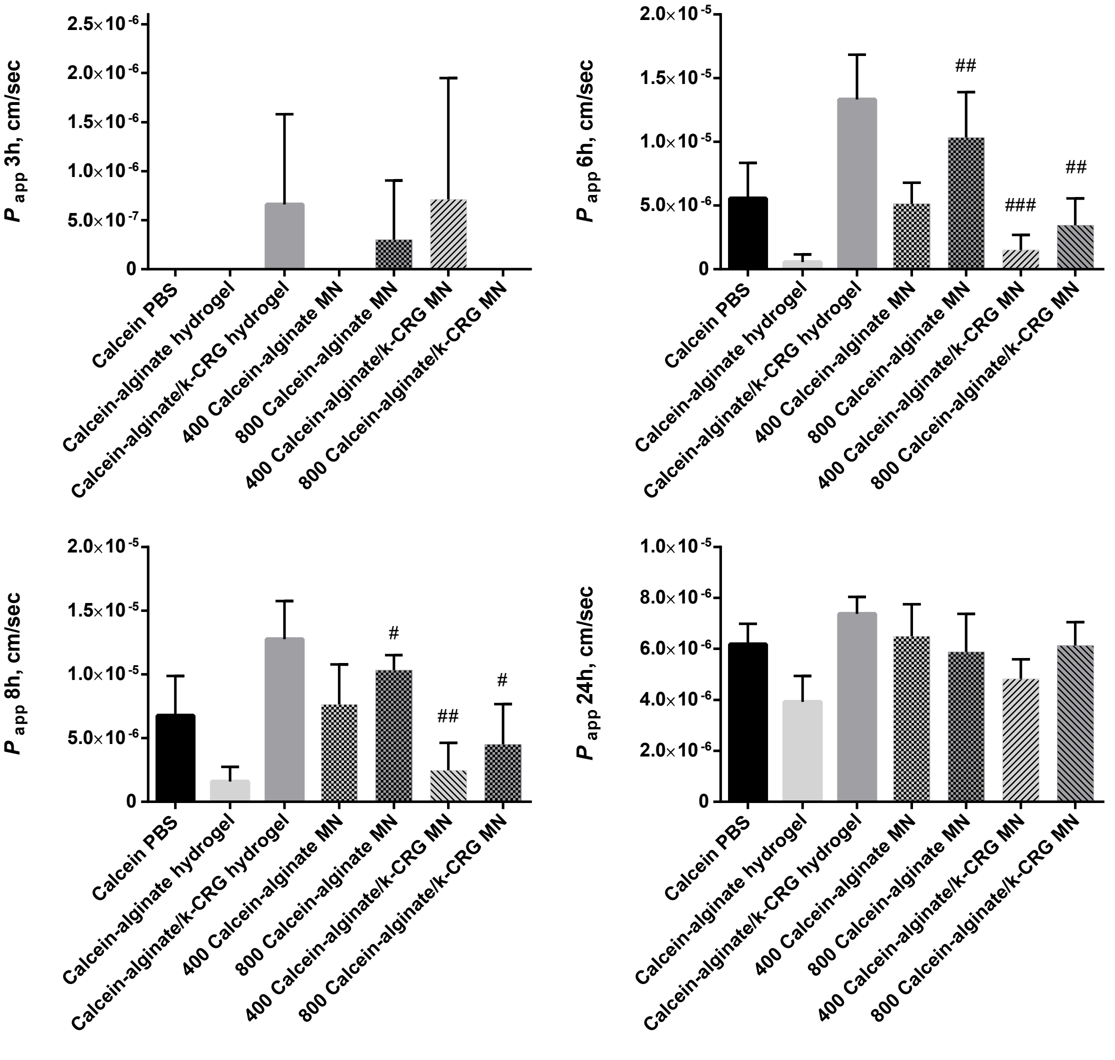

Figure S6. - Apparent permeability coefficient for 3, 6, 8, and 24 hours of calcein for porcine ear skin treated with free calcein, calcein-loaded hydrogels, and calcein-loaded MNs of two different heights. # P < 0.05 for 800 calcein-alginate MN vs hydrogel formulations at 8 hours; # P < 0.05 for 800 calcein alginate/k-CRG MN vs hydrogel formulations at 8 hours; ## P < 0.01 for 800 calcein-alginate MN and 800 alginate/k-CRG MN vs hydrogel formulations at 6 hours; ## P < 0.01 for 400 calcein alginate/k-CRG MN vs hydrogel formulation at 8 hours; ### P < 0.001 for 400 calcein alginate/k-CRG MN vs hydrogel formulations at 6 hours. Statistical significance was only represented for MN formulations vs free calcein solution or vs calcein-loaded hydrogels.

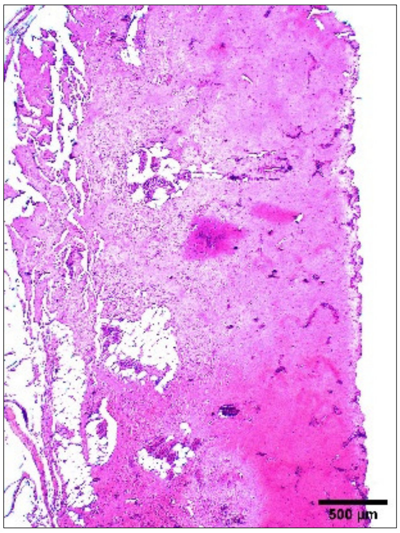

Figure S7. Optical microscopy analysis of skin sample from the permeation assay. Skin was stained with H&E upon 24 hours exposure to PBS. Scale bar 500 µm.

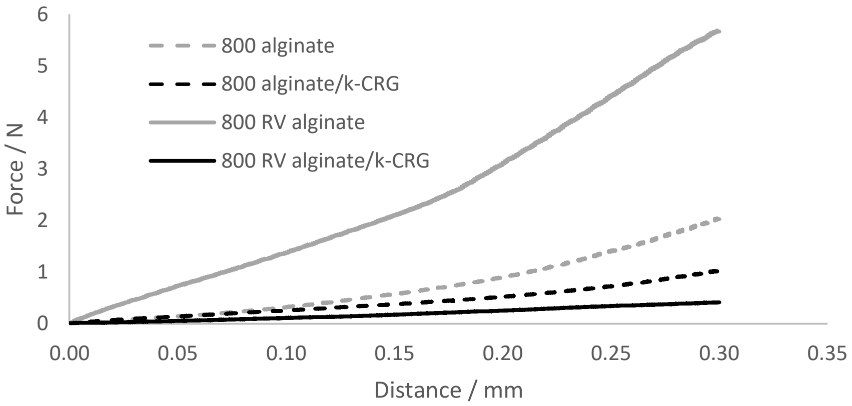

Figure S8. - Representative force *vs* displacement curves for 800 µm drug-free and RV-loaded MNs.

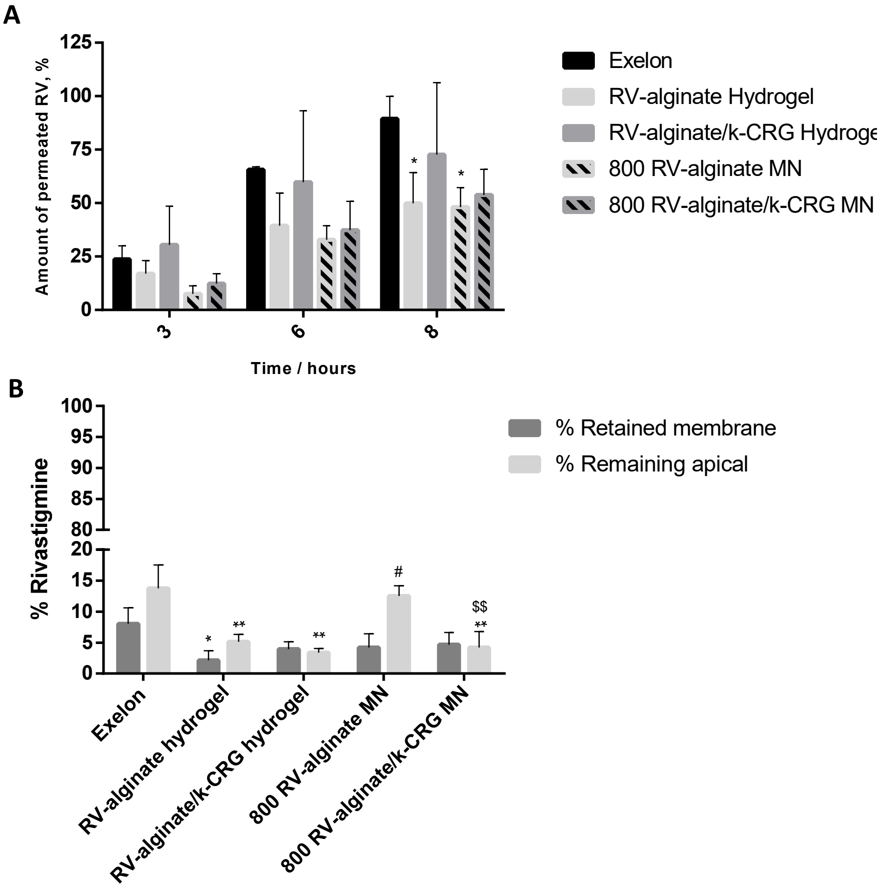

Figure S9. – Skin rivastigmine permeation profile. The bars/points represent the mean ± SD of the permeability for three independent experiments (n=3). (A) Amount of permeated RV (%) as a function of time obtained for Exelon®, RV-loaded hydrogel, and RV-loaded MNs. $^{*}P < 0.05$ for hydrogel formulation and 800 alginate MN vs Exelon® at 8 hours. (B) Distribution of RV among retained in the porcine skin and remaining in the apical comportment.** $P < 0.01$ for alginate, alginate/k-CRG hydrogel formulations and 800 RV-alginate/k-CRG MN vs Exelon® remaining in the apical; $^{\#}$ $P < 0.05$ for 800 RV-alginate MN vs hydrogel formulation remaining in the apical; $^{\$\$}$ $P < 0.01$ for 800 RV-alginate/k-CRG MN vs 800 RV-alginate MN remaining in the apical; * $P < 0.05$ for RV-alginate hydrogel formulation vs Exelon® retained in the membrane.

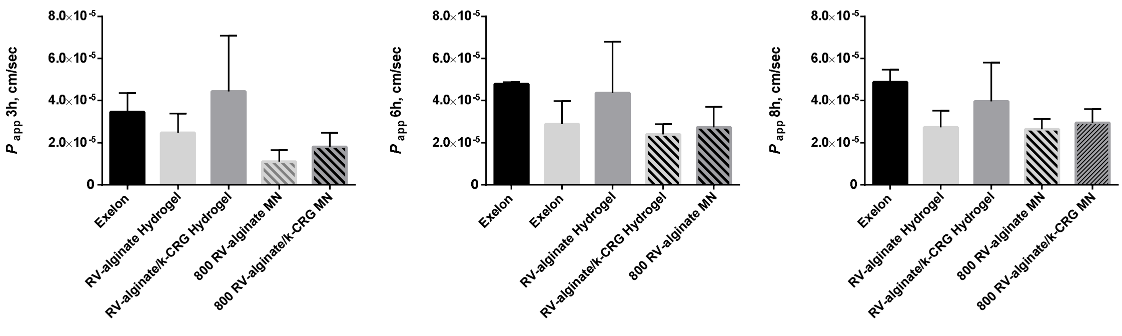

Figure S10. - Apparent permeability coefficient for 3, 6, and 8 hours of RV for porcine ear skin treated with Exelon®, RV-loaded hydrogels, and RV-loaded MNs.

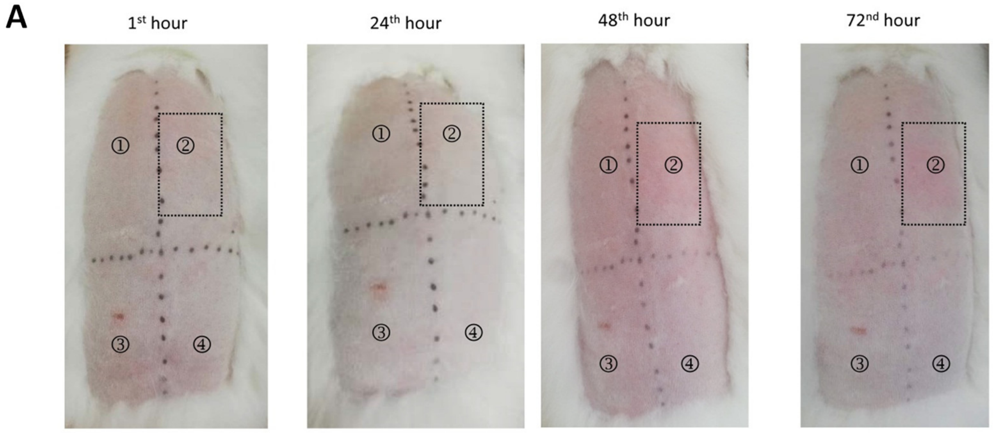

B

	Erythema				Oedema			
Time / hour	Sample Alginate MN	Sample alginate/k-CRG MN	Positive control	Exelon	Sample alginate MN	Sample alginate/k-CRG MN	Positive control	Exelon
24	0	0	1	0	0	0	0	0
48	0	0	1	0	0	0	0	0
72	0	0	2	0	0	0	0	0

Figure S11. - Skin irritation test for Alg-MN and Mix-MN after 4 hours exposition. (A) digital photography: 1 – Administered RV-alginate MN; 2 – positive control (10% SDS); 3 – Exelon® patch; 4 – administered RV-alginate/k-CRG MN. (B) Evaluation of 4 hour exposition following the scoring system for skin reaction.

Table S1. Scoring system for skin reaction.

Reaction	Score for skin reaction
Erythema and eschar formation	
No erythema	0
Very slight erythema (barely perceptible)	1
Well defined erythema	2
Moderate erythema	3
Severe erythema (beet, redness) to eschar formation preventing grading of erythema	4
Oedema formation	
No oedema	0
Very slight oedema (barely perceptible)	1
Well defined oedema (edges of area well-defined by definite raising)	2
Moderate oedema (raised approximately 1 mm)	3
Severe oedema (raised more than 1 mm and extending beyond exposure area)	4
Maximal possible score for irritation	8

Table S2. PII or CII by rabbits.

Score	Response category
**0 – 0.4**	Negligible
**0.5 – 1.9**	Slight
**2 – 4.9**	Moderate
**5 - 8**	Severe

Drug release kinetics

The following mathematical models for drug release kinetics were applied to evaluate the mechanism of drug release:

Zero order release model, refers to the process of constant drug release from a drug delivery device

$Q = Q0 + K0t$

Q: amount of drug released or dissolved

Q0: initial amount of drug in solution (it is usually zero)

K0 zero order release constant.

First order release model, drug release rate depends on its concentration

$\text{Log } C = \text{Log } C0 - kt / 2.303$

C0: initial concentration of drug

K: first order constant.

Hixson-Crowell release model, describes the release from systems where there is a change in surface area and diameter of particles.

$Q_0^{1/3} – Q_t^{1/3} = K_{HC}\, t$

Q_t: amount of drug released in time t,

Q_0: initial amount of the drug in tablet/ formulation

$K_{HC:}$ rate constant for Hixson-Crowell rate equation.

Higuchi release model, relate the drug release rate to the physical constants based on simple laws of diffusion

$Q_t = k_H (t)^{0.5}$

Qt: amount of drug released in time t,

T: time in hours

kH: release rate constant for the Higuchi model

Korsmeyer-Peppas release model,

F=Mt/M N = Ktn

F: fraction of drug release at time t

Mt / M: fraction of drug released at time t,

K: the rate constant

n: release exponent.

The model that best fits the experimental release data will be selected based on the correlation coefficient (r^2).

Table S3. Value of r^2 obtained from the release data for different models of mechanism of drug release.

Model	RV -alginate hydrogel	RV-alginate/k-CRG hydrogel	Exelon®
Zero order	**0.952**	**0.977**	**0.987**
First order	0.928	0.947	0.885
Higuchi	0.789	0.842	0.871
Hixson-Crowell	0.938	0.959	0.937
Korsmeyer-Peppas	0.856	0.899	0.924

The rivastigmine release from the hydrogels, under the different conditions studied, was diffusion controlled described by the zero-order release model, as plots of the amount released versus square root of time was found to be linear. The correlation coefficient for the different formulations was in the range between 0.952 and 0.987. Rivastigmine level at the site of action remains constant throughout the period of drug delivery once administered either as incorporated within a polymeric hydrogel or within commercial patch, independent of its concentration.
